# Supplementary material for: Inequalities in Health Care Experience of Patients with Chronic Conditions: Results from a Population-Based Study
Source: Healthcare (Basel). 2021 Aug 5;9(8):1005. doi: 10.3390/healthcare9081005 (PMC8394123; doi:10.3390/healthcare9081005)
Supplement: Supplementary file 1 [file healthcare-09-01005-s001.zip › model 1_supplementary material.pdf]

Table S1. Model 1 - WLS results (detailed). Differences in healthcare experience among patients with self-declared chronic conditions. The effect of sociodemographic and economic characteristics.

Variable	Category	Factor 1: INTER Coef. 95% C.I.	Factor 2: NEW Coef. 95% C.I.	Factor 3: SELF Coef. 95% C.I.	OVERALL IEXPAC Coef. 95% C.I.
Gender. Baseline: Women	Men	0.214**	0.022	0.168**	0.145**
		0.051,0.377	-0.106,0.150	0.009,0.326	0.017,0.273
Age ranges: Baseline: 15-24	25-44	-1.373*	-1.022	-1.080	-1.171
		-2.979,0.232	-3.229,1.184	-3.012,0.852	-2.804,0.462
	45-64	-0.808	-1.249	-0.264	-0.730
		-1.991,0.376	-3.258,0.760	-1.974,1.446	-2.151,0.690
	65-74	-0.715	-1.659	-0.338	-0.835
		-1.921,0.491	-3.663,0.345	-2.050,1.375	-2.261,0.590
	75-89	-0.097	-1.848*	0.403	-0.393
		-1.249,1.054	-3.840,0.144	-1.296,2.102	-1.793,1.007
	>=90	-1.121	-2.464**	-1.186	-1.511
		-3.518,1.275	-4.541, -0.387	-4.092,1.720	-3.661,0.639
Occupation Baseline: Managers I	Managers II	-0.355	-2.631***	-0.693	-1.098**
		-1.240,0.531	-3.733, -1.529	-1.788,0.403	-1.954, -0.243
	Intermediate	-0.660	-0.169	1.561**	0.282
		-2.127,0.807	-1.230,0.892	0.150,2.972	-0.890,1.453
	Semi-qualified	0.616	1.452*	0.974	0.974*
		-0.420,1.651	-0.053,2.956	-0.368,2.317	-0.088,2.037
	Non-qualified	0.094	-0.826	0.393	-0.048
		-0.829,1.018	-2.015,0.362	-0.757,1.543	-0.944,0.848
Education. Baseline: Primary	Secondary-lower	-1.951**	-2.070**	-2.425**	-2.156**
		-3.247, -0.656	-3.879, -0.262	-3.971, -0.879	-3.462, -0.850
	Secondary-upper	-1.041**	-0.404	-0.786	-0.775
		-2.043, -0.038	-2.157,1.348	-2.168,0.596	-1.942,0.393
	Tertiary	-0.361	-0.636	-0.656	-0.544
		-1.493,0.771	-2.814,1.543	-2.392,1.079	-1.927,0.840
Occupation#Age	Managers II # 25-44	0.849	3.679***	1.582**	1.887**
		-0.443,2.140	2.112,5.246	0.153,3.012	0.701,3.074
	Managers II # 45-64	-0.027	2.757***	0.381	0.880*
		-1.115,1.060	1.529,3.984	-0.877,1.638	-0.114,1.874
	Managers II # 65-74	-0.133	3.126***	0.612	1.027*
		-1.357,1.091	1.819,4.432	-0.753,1.977	-0.065,2.118
	Managers II # 75-89	0.349	2.859***	0.794	1.195**
		-0.818,1.515	1.531,4.188	-0.584,2.173	0.141,2.250
	Managers II # >=90	2.159*	2.454***	2.723*	2.444**
		-0.378,4.696	1.055,3.854	-0.324,5.769	0.473,4.416
	Intermediate # 25-44	-0.135	0.781	-1.891*	-0.524
		-2.071,1.802	-0.872,2.434	-3.956,0.174	-2.173,1.126
	Intermediate # 45-64	-0.311	0.088	-2.131**	-0.864
		-1.998,1.377	-1.114,1.289	-3.722, -0.540	-2.190,0.462
	Intermediate # 65-74	0.255	0.279	-1.817**	-0.492
		-1.503,2.014	-0.931,1.489	-3.498, -0.136	-1.875,0.891
	Intermediate # 75-89	0.059	0.038	-2.271**	-0.794
		-1.570,1.688	-1.193,1.270	-3.874, -0.668	-2.091,0.503
	Intermediate # >=90	2.659**	-0.104	1.279	1.404
		0.010,5.308	-1.344,1.136	-1.583,4.141	-0.643,3.450
	Semi-qualified # 25-44	-0.620	-1.062	-1.134	-0.927
		-1.925,0.684	-2.702,0.577	-2.683,0.416	-2.157,0.302
	Semi-qualified # 45-64	-0.500	-1.457*	-0.825	-0.879
		-1.643,0.643	-3.013,0.100	-2.249,0.600	-2.006,0.248
	Semi-qualified # 65-74	-0.703	-1.375*	-0.665	-0.873
		-1.901,0.495	-2.945,0.196	-2.119,0.788	-2.031,0.286

	Semi-qualified # 75-89	-1.049*	-1.643**	-1.461*	-1.361**
		-2.229,0.131	-3.199,-0.088	-2.925,0.003	-2.508,-0.214
	Semi-qualified# >=90	-0.346	-1.448	-0.204	-0.595
		-3.027,2.334	-3.191,0.294	-3.145,2.736	-2.752,1.562
	Non-qualified # 25-44	-0.136	0.903	-0.150	0.142
		-1.351,1.079	-0.404,2.211	-1.513,1.213	-0.931,1.216
	Non-qualified # 45-64	-0.327	0.772	-0.589	-0.123
		-1.362,0.707	-0.472,2.017	-1.821,0.642	-1.087,0.841
	Non-qualified # 65-74	-0.105	0.619	-0.273	0.032
		-1.194,0.984	-0.636,1.875	-1.535,0.990	-0.963,1.026
	Non-qualified # 75-89	-0.354	0.721	-0.826	-0.233
		-1.401,0.692	-0.527,1.970	-2.084,0.432	-1.205,0.740
	Non-qualified # >=90	1.061	1.604**	1.283	1.290
		-1.293,3.415	0.197,3.010	-1.348,3.914	-0.597,3.176
Education#Age	Secondary-lower # 25-44	2.070**	1.146	2.292**	1.899**
		0.320,3.821	-0.961,3.252	0.422,4.162	0.291,3.506
	Secondary-lower # 45-64	1.584**	1.261	2.002**	1.648**
		0.214,2.954	-0.581,3.104	0.399,3.606	0.295,3.001
	Secondary-lower # 65-74	1.984**	1.458	2.381**	1.985**
		0.628,3.340	-0.372,3.287	0.789,3.973	0.643,3.327
	Secondary-lower # 75-89	1.994**	1.386	2.239**	1.918**
		0.658,3.331	-0.433,3.206	0.654,3.825	0.587,3.248
	Secondary-lower # >=90	1.822**	1.638*	2.058**	1.858**
		0.162,3.483	-0.302,3.577	0.248,3.869	0.357,3.359
	Secondary-upper # 25-44	1.037	-0.362	0.555	0.480
		-0.403,2.477	-2.346,1.621	-1.074,2.185	-0.929,1.890
	Secondary-upper # 45-64	0.788	0.245	0.459	0.520
		-0.291,1.867	-1.541,2.030	-0.976,1.893	-0.691,1.731
	Secondary-upper # 65-74	1.011*	0.158	0.727	0.675
		-0.089,2.111	-1.619,1.935	-0.717,2.172	-0.541,1.892
	Secondary-upper # 75-89	1.175**	0.216	0.817	0.784
		0.078,2.273	-1.575,2.008	-0.650,2.285	-0.439,2.006
	Secondary-upper # >=90	1.796*	0.097	2.278**	1.508*
		-0.151,3.744	-1.730,1.924	0.045,4.511	-0.154,3.170
	Tertiary # 25-44	0.398	0.358	0.501	0.424
		-1.194,1.990	-2.025,2.741	-1.481,2.482	-1.199,2.047
	Tertiary # 45-64	-0.048	0.434	-0.070	0.075
		-1.299,1.203	-1.788,2.657	-1.881,1.741	-1.371,1.522
	Tertiary # 65-74	0.280	0.668	0.236	0.370
		-1.068,1.628	-1.574,2.910	-1.642,2.115	-1.129,1.869
	Tertiary # 75-89	0.044	0.679	0.194	0.272
		-1.270,1.358	-1.582,2.940	-1.688,2.076	-1.216,1.759
	Tertiary # >=90	-2.282	-0.119	-1.378	-1.363
		-7.276,2.712	-2.397,2.159	-5.781,3.026	-4.884,2.158
Constant term		8.369***	3.013**	6.936***	6.387***
		7.316,9.422	1.052,4.974	5.305,8.567	5.031,7.744
Goodness-of-fit	R-squared	0.034	0.055	0.039	0.027
	BIC	18,445.536	16,501.215	18,223.872	16,523.464
Heteroscedasticity correction method	YES	Robust variance	Robust variance	Robust variance	Robust variance
Sample size (¥)	N	3,883	3,883	3,883	3,883

* *p < 0.1*, ** *p < 0.05*, *** *p < 0.001*; Coef.: Regression coefficient; BIC: Bayesian information criterion; the presented model is corrected from heteroscedasticity using Eicker–Huber–White standard errors. ¥: Missing responses excluded for the analyses.
