# Supplementary material for: Inequalities in Health Care Experience of Patients with Chronic Conditions: Results from a Population-Based Study
Source: Healthcare (Basel). 2021 Aug 5;9(8):1005. doi: 10.3390/healthcare9081005 (PMC8394123; doi:10.3390/healthcare9081005)
Supplement: Supplementary file 1 [file healthcare-09-01005-s001.zip › model 2_supplementary material.pdf]

Table S2. Model 2—WLS results (detailed). Differences in healthcare experience among patients with self-declared chronic conditions. The effect of the presence of multi-morbidity.

Variable	Category	Factor 1: INTER Coef. 95% CI	Factor 2: NEW Coef. 95% CI	Factor 3: SELF Coef. 95% CI	OVERALL IEXPAC Coef. 95% CI
Gender	Men	0.210**	0.013	0.160**	0.138**
		0.047,0.374	-0.116,0.142	0.001,0.319	0.010,0.266
Age ranges. Baseline: 15-24	25-44	-1.493*	-1.087	-1.111	-1.243
		-3.106,0.121	-3.310,1.135	-3.080,0.858	-2.909,0.423
	45-64	-0.890	-1.201	-0.305	-0.762
		-2.072,0.292	-3.227,0.826	-2.051,1.442	-2.218,0.694
	65-74	-1.059*	-1.627	-0.608	-1.050
		-2.297,0.178	-3.656,0.401	-2.382,1.166	-2.530,0.430
	75-89	-0.020	-1.648	0.548	-0.257
		-1.189,1.150	-3.666,0.371	-1.206,2.302	-1.705,1.190
	>=90	-2.066	-3.026**	-2.399	-2.449**
		-4.685,0.553	-5.195, -0.857	-5.600,0.802	-4.760, -0.138
Education. Baseline: Primary	Secondary-lower	-2.165**	-2.225**	-2.491**	-2.300***
		-3.458, -0.873	-4.065, -0.385	-4.081, -0.901	-3.651, -0.949
	Secondary-upper	-1.169**	-0.446	-0.826	-0.847
		-2.169, -0.169	-2.189,1.297	-2.246,0.595	-2.050,0.356
	Tertiary	-0.252	-0.925	-0.703	-0.600
		-1.459,0.955	-2.923,1.072	-2.510,1.104	-1.995,0.795
Occupation Baseline: Managers I	Managers II	-0.360	-2.656***	-0.684	-1.104**
		-1.242,0.521	-3.748, -1.564	-1.792,0.424	-1.964, -0.245
	Intermediate	-0.577	-0.188	1.583**	0.315
		-2.023,0.869	-1.213,0.837	0.168,2.999	-0.846,1.475
	Semi-qualified	0.676	1.398*	0.961	0.977*
		-0.391,1.743	-0.154,2.950	-0.420,2.342	-0.119,2.072
	Non-qualified	0.303	-0.686	0.451	0.087
		-0.642,1.249	-1.854,0.481	-0.732,1.635	-0.824,0.999
Occupation#Age	Managers II # 25-44	0.932	3.836***	1.668**	1.992***
		-0.352,2.215	2.281,5.392	0.234,3.103	0.807,3.176
	Managers II # 45-64	-0.018	2.794***	0.370	0.890*
		-1.101,1.065	1.576,4.012	-0.898,1.638	-0.106,1.887
	Managers II # 65-74	-0.152	3.148***	0.587	1.017*
		-1.378,1.074	1.852,4.445	-0.793,1.967	-0.081,2.115
	Managers II # 75-89	0.353	2.829***	0.763	1.177**
		-0.813,1.520	1.498,4.160	-0.629,2.156	0.116,2.239
	Managers II # >=90	2.231	2.538***	2.778*	2.514**
		-0.512,4.974	1.126,3.951	-0.204,5.759	0.502,4.525
	Intermediate # 25-44	-0.148	0.809	-1.845*	-0.504
		-2.068,1.773	-0.837,2.455	-3.921,0.231	-2.152,1.145
	Intermediate # 45-64	-0.395	0.117	-2.161**	-0.898
		-2.063,1.273	-1.052,1.285	-3.755, -0.567	-2.213,0.418
	Intermediate # 65-74	0.193	0.272	-1.819**	-0.517
		-1.550,1.937	-0.908,1.452	-3.508, -0.129	-1.893,0.860
	Intermediate # 75-89	-0.060	0.024	-2.327**	-0.861
		-1.669,1.549	-1.172,1.219	-3.934, -0.720	-2.148,0.425
	Intermediate # >=90	2.540*	-0.125	1.225	1.335
		-0.053,5.132	-1.358,1.108	-1.633,4.083	-0.669,3.339
	Semi-qualified # 25-44	-0.581	-0.952	-1.005	-0.836
		-1.916,0.753	-2.630,0.727	-2.588,0.579	-2.095,0.422
	Semi-qualified # 45-64	-0.558	-1.395*	-0.813	-0.879
		-1.730,0.614	-2.997,0.208	-2.274,0.648	-2.038,0.280
	Semi-qualified # 65-74	-0.751	-1.335	-0.643	-0.871
		-1.976,0.474	-2.952,0.282	-2.131,0.845	-2.060,0.318
	Semi-qualified # 75-89	-1.118*	-1.591*	-1.461*	-1.372**
		-2.326,0.090	-3.192,0.009	-2.960,0.038	-2.550, -0.194

	Semi-qualified # >=90	-0.223	-1.306	0.043	-0.422
		-2.927,2.481	-3.114,0.501	-2.918,3.003	-2.593,1.749
	Non-qualified # 25-44	-0.280	0.819	-0.131	0.074
		-1.518,0.959	-0.468,2.105	-1.525,1.264	-1.015,1.163
	Non-qualified # 45-64	-0.528	0.646	-0.647	-0.251
		-1.583,0.526	-0.580,1.871	-1.910,0.617	-1.230,0.727
	Non-qualified # 65-74	-0.323	0.473	-0.341	-0.112
		-1.430,0.785	-0.762,1.709	-1.633,0.952	-1.121,0.896
	Non-qualified # 75-89	-0.573	0.563	-0.908	-0.385
		-1.640,0.493	-0.664,1.790	-2.197,0.381	-1.371,0.600
	Non-qualified # >=90	1.077	1.556**	1.514	1.367
		-1.302,3.457	0.154,2.959	-1.146,4.174	-0.530,3.264
Education#Age	Secondary-lower # 25-44	2.297**	1.220	2.331**	2.016**
		0.538,4.057	-0.919,3.359	0.419,4.243	0.369,3.663
	Secondary-lower # 45-64	1.855**	1.478	2.107**	1.844**
		0.485,3.224	-0.396,3.352	0.459,3.755	0.446,3.241
	Secondary-lower # 65-74	2.054**	1.660*	2.322**	2.044**
		0.698,3.410	-0.200,3.521	0.685,3.959	0.658,3.431
	Secondary-lower # 75-89	2.182**	1.667*	2.323**	2.093**
		0.842,3.521	-0.184,3.518	0.689,3.957	0.715,3.470
	Secondary-lower # >=90	1.814**	1.758*	1.911**	1.834**
		0.146,3.482	-0.238,3.754	0.058,3.764	0.281,3.387
	Secondary-upper # 25-44	1.174	-0.332	0.612	0.559
		-0.267,2.615	-2.306,1.642	-1.049,2.273	-0.878,1.995
	Secondary-upper # 45-64	0.926*	0.292	0.509	0.601
		-0.151,2.003	-1.485,2.069	-0.964,1.981	-0.644,1.847
	Secondary-upper # 65-74	1.109**	0.206	0.738	0.728
		0.013,2.205	-1.562,1.974	-0.742,2.219	-0.522,1.978
	Secondary-upper # 75-89	1.309**	0.307	0.877	0.879
		0.214,2.404	-1.474,2.088	-0.627,2.380	-0.378,2.135
	Secondary-upper # >=90	1.589	0.017	1.976*	1.301
		-0.331,3.509	-1.855,1.889	-0.258,4.210	-0.365,2.966
	Tertiary # 25-44	0.251	0.584	0.507	0.435
		-1.401,1.903	-1.636,2.804	-1.537,2.552	-1.199,2.068
	Tertiary # 45-64	-0.164	0.703	-0.019	0.125
		-1.485,1.156	-1.343,2.748	-1.899,1.861	-1.334,1.583
	Tertiary # 65-74	0.156	0.950	0.272	0.415
		-1.257,1.569	-1.117,3.016	-1.674,2.217	-1.097,1.926
	Tertiary # 75-89	-0.071	0.950	0.218	0.312
		-1.458,1.315	-1.140,3.039	-1.733,2.169	-1.190,1.815
	Tertiary # >=90	-1.987	0.226	-1.042	-1.040
		-7.413,3.439	-1.966,2.418	-5.755,3.671	-4.867,2.787
Number of conditions	2	-0.636	0.286	-0.068	-0.178
Baseline: 1		-1.569,0.298	-0.961,1.533	-1.055,0.919	-0.930,0.574
	3	-1.528*	-1.870***	-0.520	-1.255**
		-3.266,0.209	-2.743,-0.997	-1.703,0.662	-2.240,-0.269
	+3	0.341	0.307	0.322	0.325
		-0.803,1.485	-0.969,1.584	-0.985,1.629	-0.303,0.953
Age#Number of diseases	25-44 # 2	0.506	-0.652	-0.105	-0.032
		-0.597,1.608	-1.982,0.679	-1.223,1.012	-0.903,0.839
	25-44 # 3	1.472	2.258***	0.441	1.312**
		-0.508,3.452	0.992,3.523	-1.059,1.942	0.054,2.569
	25-44 # +3	-0.840	-0.185	-0.697	-0.609
		-2.316,0.636	-1.612,1.241	-2.250,0.857	-1.558,0.339
	45-64 # 2	0.522	-0.514	0.053	0.069
		-0.474,1.518	-1.792,0.763	-0.987,1.094	-0.731,0.869
	45-64 # 3	1.634*	1.727***	0.782	1.349**
		-0.155,3.423	0.769,2.685	-0.471,2.034	0.305,2.393
	45-64 # +3	-0.693	-0.740	-0.523	-0.644*
		-1.900,0.514	-2.053,0.572	-1.879,0.832	-1.339,0.051
	65-74 # 2	0.884*	-0.312	0.414	0.387
		-0.165,1.933	-1.599,0.975	-0.673,1.502	-0.452,1.226

	65-74 # 3	1.686*	1.601***	0.672	1.294**
		-0.121,3.493	0.668,2.534	-0.601,1.944	0.239,2.349
	65-74 # +3	0.310	-0.515	0.311	0.085
		-0.905,1.526	-1.829,0.799	-1.057,1.678	-0.618,0.789
	75-89 # 2	0.391	-0.360	0.057	0.065
		-0.649,1.432	-1.659,0.939	-1.039,1.153	-0.773,0.902
	75-89 # 3	1.125	1.517**	0.102	0.860
		-0.680,2.931	0.576,2.458	-1.185,1.390	-0.199,1.920
	75-89 # +3	-0.508	-0.839	-0.536	-0.608*
		-1.716,0.701	-2.148,0.471	-1.909,0.837	-1.308,0.092
	>=90 # 2	0.873	0.036	0.772	0.608
		-1.300,3.047	-1.453,1.525	-1.425,2.969	-0.995,2.212
	>=90 # 3	2.209*	2.554***	1.673	2.108**
		-0.277,4.695	1.259,3.849	-0.618,3.964	0.433,3.783
	>=90 # +3	0.820	0.239	1.167	0.788
		-0.846,2.486	-1.239,1.717	-0.735,3.068	-0.322,1.898
Constant term		8.506***	3.089**	6.975***	6.472***
		7.465,9.547	1.114,5.064	5.312,8.638	5.083,7.861
Goodness-of-fit	R-squared	0.039	0.063	0.044	0.033
	BIC	18,549.184	16,589.597	18,324.712	16,624.089
Heteroscedasticity correction method	YES	Robust variance	Robust variance	Robust variance	Robust variance
Sample size (¥)	N	3,878	3,878	3,878	3,878

* $p < 0.1$, ** $p < 0.05$, *** $p < 0.001$; Coef.: Regression coefficient; BIC: Bayesian information criterion; the presented model is corrected from heteroscedasticity using Eicker–Huber–White standard errors. ¥: Missing responses excluded for the analyses.
