# Supplementary material for: Inequalities in Health Care Experience of Patients with Chronic Conditions: Results from a Population-Based Study
Source: Healthcare (Basel). 2021 Aug 5;9(8):1005. doi: 10.3390/healthcare9081005 (PMC8394123; doi:10.3390/healthcare9081005)
Supplement: Supplementary file 1 [file healthcare-09-01005-s001.zip › model 3_supplementary material.pdf]

Table S3. Model 3—WLS results (detailed). Differences in healthcare experience among patients with self-declared chronic conditions. The effect of chronic conditions.

**Variable**	**Category**	**Factor 1: INTER Coef. 95% C.I.**	**Factor 2: NEW Coef. 95% C.I.**	**Factor 3: SELF Coef. 95% C.I.**	**OVERALL IEXPAC Coef. 95% C.I.**
Gender	Men	0.075	0.013	0.059	0.052
		-0.104,0.253	-0.127,0.152	-0.115,0.232	-0.087,0.191
Age ranges. Baseline: 15-24	25-44	-1.220	-0.888	-0.942	-1.028
		-2.908,0.467	-3.221,1.445	-2.998,1.115	-2.787,0.731
	45-64	-0.705	-1.063	-0.163	-0.606
		-1.995,0.585	-3.210,1.084	-2.017,1.690	-2.171,0.959
	65-74	-0.787	-1.469	-0.423	-0.841
		-2.106,0.532	-3.611,0.673	-2.286,1.441	-2.416,0.735
	75-89	-0.164	-1.644	0.357	-0.378
		-1.426,1.097	-3.773,0.486	-1.486,2.200	-1.924,1.168
	>=90	-1.160	-2.141*	-1.251	-1.460
		-3.631,1.311	-4.440,0.159	-4.258,1.756	-3.749,0.828
Occupation Baseline: Managers I	Managers II	-0.136	-2.470***	-0.537	-0.918**
		-1.063,0.791	-3.605,-1.334	-1.676,0.603	-1.808,-0.028
	Intermediate	-0.520	-0.171	1.725**	0.392
		-2.167,1.128	-1.280,0.937	0.085,3.365	-0.946,1.729
	Semi-qualified	0.739	1.324*	1.011	0.998*
		-0.342,1.821	-0.218,2.867	-0.398,2.419	-0.111,2.106
	Non-qualified	0.174	-0.779	0.424	0.005
		-0.767,1.115	-2.003,0.446	-0.754,1.603	-0.922,0.933
Education. Baseline: Primary	Secondary-lower	-1.774**	-1.849*	-2.169**	-1.938**
		-3.128, -0.419	-3.730,0.031	-3.797, -0.541	-3.329, -0.546
	Secondary-upper	-0.871	-0.180	-0.525	-0.557
		-1.945,0.202	-2.013,1.653	-1.990,0.940	-1.819,0.705
	Tertiary	-0.126	-0.270	-0.240	-0.207
		-1.374,1.122	-2.541,2.000	-2.074,1.594	-1.707,1.293
Occupation#Age	Managers II # 25-44	0.621	3.508***	1.418*	1.698**
		-0.683,1.925	1.920,5.097	-0.023,2.859	0.510,2.886
	Managers II # 45-64	-0.278	2.573***	0.180	0.666
		-1.399,0.842	1.320,3.827	-1.116,1.476	-0.356,1.688
	Managers II # 65-74	-0.392	2.923***	0.422	0.808
		-1.645,0.860	1.591,4.256	-0.981,1.826	-0.312,1.928
	Managers II # 75-89	0.228	2.663***	0.707	1.066*
		-0.976,1.432	1.302,4.024	-0.710,2.125	-0.015,2.148
	Managers II # >=90	2.089	2.145**	2.507	2.256**
		-0.542,4.719	0.485,3.804	-0.560,5.573	0.226,4.286
	Intermediate # 25-44	-0.083	0.877	-1.881*	-0.475
		-2.142,1.976	-0.830,2.583	-4.113,0.352	-2.241,1.291
	Intermediate # 45-64	-0.460	0.081	-2.322**	-0.990
		-2.305,1.385	-1.156,1.318	-4.117,-0.528	-2.461,0.482
	Intermediate # 65-74	0.126	0.154	-2.025**	-0.649
		-1.796,2.048	-1.100,1.408	-3.905,-0.146	-2.180,0.882
	Intermediate # 75-89	-0.066	-0.009	-2.452**	-0.918
		-1.864,1.731	-1.297,1.280	-4.275,-0.628	-2.380,0.544
	Intermediate # >=90	2.687**	-0.190	1.158	1.346
		0.001,5.372	-1.814,1.434	-1.959,4.276	-0.900,3.593
	Semi-qualified # 25-44	-0.748	-0.923	-1.143	-0.939
		-2.097,0.600	-2.597,0.751	-2.749,0.463	-2.210,0.331
	Semi-qualified # 45-64	-0.687	-1.368*	-0.939	-0.964
		-1.871,0.497	-2.961,0.225	-2.423,0.546	-2.133,0.205
	Semi-qualified # 65-74	-0.836	-1.278	-0.688	-0.903
		-2.075,0.402	-2.887,0.331	-2.204,0.829	-2.106,0.300
	Semi-qualified # 75-89	-1.199*	-1.580*	-1.565**	-1.436**
		-2.417,0.020	-3.177,0.017	-3.094, -0.035	-2.628, -0.243
	Semi-qualified # >=90	-0.347	-1.481	-0.104	-0.567
		-3.068,2.375	-3.378,0.417	-3.094,2.887	-2.792,1.657
	Non-qualified # 25-44	-0.249	0.850	-0.202	0.068
		-1.479,0.981	-0.491,2.192	-1.585,1.180	-1.031,1.166
	Non-qualified # 45-64	-0.458	0.663	-0.706	-0.242
		-1.508,0.593	-0.616,1.942	-1.966,0.554	-1.236,0.752
	Non-qualified # 65-74	-0.166	0.557	-0.271	-0.007

		-1.270,0.937	-0.734,1.849	-1.562,1.020	-1.032,1.018
	Non-qualified # 75-89	-0.436	0.663	-0.883	-0.299
		-1.498,0.626	-0.621,1.947	-2.169,0.403	-1.301,0.703
	Non-qualified # >=90	0.981	1.444*	1.258	1.208
		-1.387,3.350	-0.130,3.019	-1.401,3.918	-0.738,3.154
Education#Age	Secondary-lower # 25-44	1.931**	0.991	2.130**	1.747**
		0.138,3.725	-1.185,3.168	0.185,4.075	0.067,3.428
	Secondary-lower # 45-64	1.474**	1.187	1.884**	1.545**
		0.048,2.901	-0.726,3.101	0.202,3.566	0.110,2.980
	Secondary-lower # 65-74	1.868**	1.464	2.257**	1.899**
		0.449,3.286	-0.436,3.365	0.582,3.932	0.472,3.327
	Secondary-lower # 75-89	1.868**	1.456	2.110**	1.844**
		0.469,3.268	-0.435,3.347	0.442,3.777	0.428,3.259
	Secondary-lower # >=90	1.658*	1.728*	2.078**	1.830**
		-0.079,3.395	-0.320,3.776	0.164,3.992	0.222,3.437
	Secondary-upper # 25-44	0.961	-0.504	0.440	0.372
		-0.526,2.448	-2.561,1.554	-1.258,2.137	-1.114,1.858
	Secondary-upper # 45-64	0.714	0.102	0.330	0.407
		-0.431,1.858	-1.764,1.968	-1.184,1.844	-0.895,1.710
	Secondary-upper # 65-74	0.974	-0.004	0.606	0.573
		-0.190,2.138	-1.861,1.853	-0.917,2.128	-0.733,1.880
	Secondary-upper # 75-89	1.112*	0.075	0.660	0.665
		-0.052,2.276	-1.796,1.947	-0.886,2.206	-0.650,1.980
	Secondary-upper # >=90	1.614	-0.029	1.858	1.255
		-0.321,3.549	-1.980,1.922	-0.410,4.126	-0.454,2.963
	Tertiary # 25-44	0.233	0.093	0.218	0.189
		-1.440,1.905	-2.376,2.562	-1.846,2.282	-1.531,1.910
	Tertiary # 45-64	-0.247	0.084	-0.418	-0.219
		-1.602,1.108	-2.229,2.397	-2.324,1.488	-1.777,1.339
	Tertiary # 65-74	0.092	0.399	-0.066	0.118
		-1.358,1.542	-1.930,2.728	-2.038,1.906	-1.491,1.727
	Tertiary # 75-89	-0.091	0.334	-0.171	-0.004
		-1.508,1.325	-2.017,2.685	-2.143,1.802	-1.602,1.593
	Tertiary # >=90	-2.523	-0.520	-1.659	-1.663
		-7.555,2.508	-2.954,1.914	-6.134,2.817	-5.281,1.956
Chronic conditions	Hypertension	0.109	-0.027	0.160*	0.090
		-0.069,0.287	-0.163,0.110	-0.012,0.332	-0.048,0.229
	Cholesterol (high)	0.144	-0.102	0.123	0.069
		-0.033,0.320	-0.238,0.034	-0.050,0.295	-0.068,0.206
	Osteoarthritis	-0.119	-0.158*	-0.007	-0.089
		-0.354,0.117	-0.317,0.000	-0.234,0.220	-0.265,0.088
	Lower-back pain	-0.094	-0.269**	-0.184	-0.174*
		-0.332,0.144	-0.448, -0.091	-0.422,0.054	-0.357,0.008
	Diabetes	0.485***	0.120	0.521***	0.398***
		0.276,0.694	-0.062,0.301	0.313,0.728	0.233,0.564
	Other cardiovascular	0.021	0.063	0.107	0.064
		-0.237,0.279	-0.126,0.251	-0.156,0.370	-0.140,0.268
	Neck pain	0.015	-0.169*	-0.240*	-0.128
		-0.260,0.289	-0.368,0.030	-0.510,0.031	-0.332,0.076
	Thyroids	-0.009	-0.009	0.060	0.016
		-0.307,0.289	-0.225,0.206	-0.212,0.332	-0.209,0.241
	Insomnia	-0.079	-0.078	-0.095	-0.085
		-0.407,0.248	-0.275,0.119	-0.394,0.204	-0.326,0.157
	Allergy	-0.146	-0.245**	-0.113	-0.161
		-0.462,0.170	-0.475, -0.016	-0.420,0.194	-0.403,0.081
	Asthma	0.216	0.230	0.232	0.226*
		-0.101,0.534	-0.046,0.507	-0.074,0.538	-0.030,0.482
	Deafness	0.155	-0.151	0.132	0.063
		-0.133,0.444	-0.366,0.064	-0.159,0.422	-0.156,0.282
	Osteoporosis	-0.175	-0.039	-0.144	-0.127
		-0.510,0.161	-0.267,0.189	-0.472,0.184	-0.387,0.134
	Cardiovascular	0.203	-0.129	-0.112	-0.002
		-0.106,0.513	-0.363,0.104	-0.413,0.190	-0.231,0.227
	Varicose veins (legs)	0.198	-0.259**	0.113	0.042
		-0.100,0.496	-0.458,-0.060	-0.176,0.402	-0.173,0.258
	Other	-0.030	0.024	0.093	0.030
		-0.387,0.328	-0.266,0.315	-0.243,0.430	-0.241,0.301
	Skin conditions	-0.306	-0.287**	-0.351**	-0.317**
		-0.684,0.071	-0.513,-0.060	-0.700,-0.003	-0.591,-0.044
	Depression	0.036	0.057	0.188	0.097

		-0.363,0.436	-0.264,0.378	-0.218,0.595	-0.228,0.422
	Anxiety	-0.289	0.228	-0.017	-0.049
		-0.705,0.127	-0.087,0.542	-0.428,0.395	-0.376,0.278
	Migraine	-0.043	-0.110	-0.101	-0.082
		-0.422,0.337	-0.365,0.145	-0.466,0.263	-0.365,0.201
	Other mouth	-0.528**	0.036	-0.158	-0.239
		-0.906, -0.149	-0.296,0.369	-0.525,0.210	-0.533,0.054
	Caries	0.075	-0.163	-0.165	-0.077
		-0.308,0.458	-0.437,0.110	-0.534,0.203	-0.361,0.207
	Peptic Ulcer conditions	-0.441*	0.179	-0.297	-0.220
		-0.898,0.017	-0.148,0.505	-0.729,0.135	-0.566,0.127
	Prostate	0.311*	-0.280*	0.146	0.090
		-0.015,0.638	-0.579,0.019	-0.233,0.526	-0.187,0.367
	Hemorrhoids	-0.141	0.033	0.099	-0.006
		-0.525,0.244	-0.278,0.345	-0.296,0.495	-0.306,0.295
	Cancer	0.254	0.742***	0.504**	0.478**
		-0.201,0.709	0.301,1.183	0.029,0.980	0.084,0.872
	Cataracts	0.053	-0.031	0.009	0.014
		-0.354,0.459	-0.321,0.260	-0.402,0.421	-0.283,0.311
	COPD	0.060	0.034	0.206	0.106
		-0.479,0.598	-0.354,0.421	-0.274,0.687	-0.306,0.518
	Incontinence	-0.200	-0.024	-0.247	-0.169
		-0.696,0.295	-0.432,0.384	-0.731,0.237	-0.547,0.209
	Kidney conditions	-0.310	0.162	-0.056	-0.089
		-0.853,0.233	-0.204,0.528	-0.559,0.446	-0.494,0.317
	Blindness	-0.208	-0.043	-0.070	-0.113
		-0.720,0.303	-0.416,0.330	-0.552,0.412	-0.500,0.274
	Dementia	0.017	0.322	-0.066	0.070
		-0.549,0.583	-0.086,0.731	-0.684,0.552	-0.399,0.539
	Constipation	-0.325	-0.144	-0.609**	-0.379**
		-0.792,0.143	-0.417,0.130	-1.058, -0.160	-0.710, -0.048
	Anemia	0.556**	0.212	0.523**	0.450**
		0.133,0.980	-0.190,0.613	0.067,0.979	0.107,0.793
	Thrombosis	0.292	-0.036	0.649**	0.333
		-0.263,0.848	-0.389,0.318	0.116,1.182	-0.078,0.743
	Other mental	0.550**	0.548*	0.773**	0.631**
		0.073,1.027	-0.049,1.145	0.207,1.340	0.172,1.089
	Fibromyalgia	0.154	0.878**	0.612**	0.518**
		-0.507,0.815	0.301,1.455	0.047,1.176	0.011,1.025
	AMI	0.118	0.221	0.094	0.137
		-0.455,0.691	-0.291,0.734	-0.508,0.695	-0.329,0.604
	Diabetic foot	-0.180	0.780	0.391	0.289
		-1.263,0.903	-0.411,1.971	-1.165,1.946	-0.860,1.439
Constant term		8.266***	2.882**	6.741***	6.243***
		7.093,9.438	0.782,4.983	4.960,8.523	4.735,7.751
Goodness-of-fit	R-squared	0.051	0.082	0.061	0.049
	BIC	18,697.108	16,711.820	18,455.281	16,755.162
Heteroscedasticity correction method	YES	Robust variance	Robust variance	Robust variance	Robust variance
Sample size (¥)	N	3,883	3,883	3,883	3,883

* $p < 0.1$, ** $p < 0.05$, *** $p < 0.001$; Coef.: Regression coefficient; COPD: chronic obstructive pulmonary disease; AMI: acute myocardial infarction. BIC: Bayesian information criterion; the presented model is corrected from heteroscedasticity using Eicker–Huber–White standard errors. ¥: Missing responses excluded for the analyses.
