# Supplementary material for: Inequalities in Health Care Experience of Patients with Chronic Conditions: Results from a Population-Based Study
Source: Healthcare (Basel). 2021 Aug 5;9(8):1005. doi: 10.3390/healthcare9081005 (PMC8394123; doi:10.3390/healthcare9081005)
Supplement: Supplementary file 1 [file healthcare-09-01005-s001.zip › model 4_supplementary_material.pdf]

Table S4. Model 4—WLS results (detailed). Differences in healthcare experience among patients with self-declared chronic conditions. The effect of self-reported Quality of life.

Independent variables	Category	Factor 1: INTER Coef. 95% CI	Factor 2: NEW Coef. 95% CI	Factor 3: SELF Coef. 95% CI	OVERALL IEXPAC Coef. 95% CI
Gender	Men	0.141*	0.012	0.178**	0.119*
		-0.019,0.300	-0.117,0.141	0.014,0.342	-0.009,0.248
Age ranges. Baseline: 15-24	25-44	-1.022	-1.000	-1.297	-1.116
		-2.936,0.893	-3.202,1.203	-2.871,0.278	-2.729,0.498
	45-64	-0.225	-1.233	-0.756	-0.693
		-1.921,1.472	-3.239,0.772	-1.920,0.408	-2.100,0.714
	65-74	-0.311	-1.649	-0.680	-0.810
		-2.011,1.389	-3.648,0.351	-1.868,0.508	-2.222,0.602
	75-89	0.494	-1.812*	0.022	-0.306
		-1.194,2.182	-3.800,0.176	-1.116,1.160	-1.695,1.082
	>=90	-0.988	-2.386**	-0.861	-1.323
		-3.838,1.862	-4.457, -0.315	-3.184,1.462	-3.419,0.773
Occupation Baseline: Managers I	Managers II	-0.700	-2.634***	-0.365	-1.106**
		-1.787,0.386	-3.734, -1.534	-1.241,0.511	-1.953, -0.259
	Intermediate	1.570**	-0.166	-0.649	0.290
		0.159,2.980	-1.226,0.895	-2.123,0.825	-0.883,1.463
	Semi-qualified	0.999	1.461*	0.647	0.997
		-0.334,2.331	-0.042,2.964	-0.384,1.679	-0.059,2.053
	Non-qualified	0.399	-0.824	0.102	-0.043
		-0.742,1.539	-2.011,0.363	-0.809,1.013	-0.930,0.844
Education. Baseline: Primary	Secondary-lower	-2.427**	-2.071**	-1.954**	-2.158**
		-3.965, -0.889	-3.879, -0.264	-3.238, -0.671	-3.457,-0.860
	Secondary-upper	-0.791	-0.406	-1.047**	-0.779
		-2.168,0.587	-2.158,1.345	-2.031, -0.063	-1.939,0.381
	Tertiary	-0.670	-0.641	-0.379	-0.556
		-2.395,1.055	-2.819,1.537	-1.489,0.732	-1.930,0.818
Occupation#Age	Managers II # 25-44	1.588**	3.681***	0.857	1.893**
		0.169,3.008	2.116,5.247	-0.423,2.136	0.716,3.070
	Managers II # 45-64	0.382	2.757***	-0.026	0.882*
		-0.867,1.631	1.532,3.982	-1.105,1.053	-0.104,1.868
	Managers II # 65-74	0.627	3.131***	-0.114	1.040*
		-0.729,1.982	1.827,4.436	-1.328,1.099	-0.043,2.123
	Managers II # 75-89	0.813	2.867***	0.373	1.213**
		-0.558,2.183	1.540,4.193	-0.788,1.533	0.165,2.260
	Managers II # >=90	2.802*	2.486***	2.263*	2.520**
		-0.300,5.903	1.110,3.861	-0.291,4.817	0.524,4.515
	Intermediate # 25-44	-1.867*	0.790	-0.103	-0.501
		-3.918,0.184	-0.859,2.439	-2.025,1.818	-2.137,1.135
	Intermediate # 45-64	-2.130**	0.088	-0.309	-0.863
		-3.720, -0.539	-1.113,1.289	-2.003,1.385	-2.190,0.465
	Intermediate # 65-74	-1.819**	0.278	0.252	-0.494
		-3.502, -0.137	-0.933,1.489	-1.515,2.019	-1.881,0.893
	Intermediate # 75-89	-2.285**	0.033	0.040	-0.807
		-3.891, -0.680	-1.200,1.266	-1.600,1.681	-2.110,0.495
	Intermediate # >=90	1.281	-0.103	2.661**	1.406
		-1.458,4.021	-1.329,1.122	0.092,5.231	-0.549,3.360
	Semi-qualified # 25-44	-1.151	-1.069	-0.643	-0.944
		-2.690,0.389	-2.707,0.569	-1.941,0.655	-2.165,0.277
	Semi-qualified # 45-64	-0.843	-1.464*	-0.525	-0.897
		-2.258,0.571	-3.019,0.091	-1.663,0.614	-2.017,0.223
	Semi-qualified # 65-74	-0.695	-1.387*	-0.743	-0.901
		-2.140,0.749	-2.956,0.183	-1.939,0.453	-2.054,0.252
	Semi-qualified # 75-89	-1.495**	-1.657**	-1.094*	-1.393**
		-2.951, -0.040	-3.211, -0.102	-2.273,0.085	-2.535, -0.252
	Semi-qualified # >=90	-0.237	-1.461*	-0.389	-0.626
		-3.131,2.658	-3.199,0.277	-3.026,2.247	-2.744,1.492
	Non-qualified # 25-44	-0.159	0.900	-0.148	0.134
		-1.512,1.195	-0.406,2.206	-1.350,1.053	-0.930,1.198
	Non-qualified # 45-64	-0.593	0.771	-0.333	-0.126
		-1.815,0.629	-0.472,2.014	-1.355,0.690	-1.081,0.828
	Non-qualified # 65-74	-0.283	0.615	-0.119	0.021
		-1.537,0.970	-0.639,1.869	-1.198,0.960	-0.965,1.008

	Non-qualified # 75-89	-0.840	0.716	-0.372	-0.246
		-2.090,0.410	-0.531,1.963	-1.411,0.666	-1.210,0.719
	Non-qualified # >=90	1.234	1.584**	0.997	1.243
		-1.333,3.802	0.185,2.984	-1.277,3.270	-0.582,3.068
Education#Age	Secondary-lower # 25-44	2.265**	1.135	2.035**	1.873**
		0.406,4.124	-0.970,3.240	0.304,3.766	0.278,3.469
	Secondary-lower # 45-64	2.015**	1.266	1.601**	1.660**
		0.419,3.611	-0.574,3.107	0.243,2.959	0.315,3.005
	Secondary-lower # 65-74	2.413**	1.470	2.026**	2.015**
		0.828,3.998	-0.358,3.299	0.680,3.371	0.680,3.350
	Secondary-lower # 75-89	2.263**	1.395	2.025**	1.940**
		0.685,3.841	-0.423,3.214	0.700,3.350	0.617,3.263
	Secondary-lower # >=90	2.109**	1.658*	1.889**	1.906**
		0.304,3.915	-0.284,3.599	0.238,3.540	0.409,3.403
	Secondary-upper # 25-44	0.523	-0.375	0.994	0.449
		-1.096,2.142	-2.356,1.606	-0.418,2.406	-0.946,1.844
	Secondary-upper # 45-64	0.453	0.242	0.781	0.515
		-0.976,1.883	-1.542,2.027	-0.280,1.842	-0.689,1.718
	Secondary-upper # 65-74	0.742	0.164	1.031*	0.689
		-0.698,2.182	-1.613,1.940	-0.053,2.114	-0.520,1.898
	Secondary-upper # 75-89	0.813	0.215	1.170**	0.779
		-0.650,2.275	-1.576,2.005	0.088,2.251	-0.436,1.995
	Secondary-upper # >=90	2.227*	0.077	1.729*	1.460*
		-0.006,4.460	-1.746,1.900	-0.211,3.669	-0.197,3.116
	Tertiary # 25-44	0.462	0.342	0.347	0.388
		-1.504,2.428	-2.040,2.724	-1.214,1.909	-1.220,1.995
	Tertiary # 45-64	-0.080	0.431	-0.061	0.066
		-1.880,1.721	-1.791,2.653	-1.292,1.170	-1.371,1.503
	Tertiary # 65-74	0.239	0.669	0.283	0.373
		-1.629,2.107	-1.573,2.911	-1.045,1.612	-1.117,1.862
	Tertiary # 75-89	0.172	0.670	0.015	0.251
		-1.701,2.045	-1.591,2.931	-1.283,1.312	-1.229,1.730
	Tertiary # >=90	-1.374	-0.118	-2.277	-1.359
		-5.720,2.973	-2.383,2.148	-7.155,2.602	-4.807,2.088
HRQoL	EQ-5Dindex	0.563**	0.222	0.742**	0.535**
		0.112,1.015	-0.067,0.511	0.292,1.192	0.186,0.884
Constant term	Constant	6.412***	2.807**	7.679***	5.890***
		4.743,8.082	0.835,4.779	6.562,8.796	4.510,7.269
Goodness-of-fit	R-squared	0.040	0.056	0.037	0.029
	BIC	18,224.702	16,507.682	18,441.584	16,521.305
Heteroscedasticity correction method	YES	Robust variance	Robust variance	Robust variance	Robust variance
Sample size	N	3,883	3,883	3,883	3,883

* $p < 0.1$, ** $p < 0.05$, *** $p < 0.001$; Coef.: Regression coefficient; QoL: Quality of Life; BIC: Bayesian information criterion; the presented model is corrected from heteroscedasticity using Eicker–Huber–White standard errors. ¥: Missing responses excluded for the analyses.
